# The functions and prognostic values of m6A RNA methylation regulators in thyroid carcinoma

**DOI:** 10.1186/s12935-021-02090-9

**Published:** 2021-07-19

**Authors:** Zhi-Hao Yu, Shao-Ting Feng, Di Zhang, Xu-Chen Cao, Yue Yu, Xin Wang

**Affiliations:** 1 The First Department of Breast Cancer, Tianjin Medical University Cancer Institute and Hospital, National Clinical Research Center for Cancer, Huan-Hu-Xi Road, He-Xi District, Tianjin, 300060 China; 2 Key Laboratory of Cancer Prevention and Therapy, Tianjin, 300060 China; 3 Tianjin’s Clinical Research Center for Cancer, Tianjin, 300060 China; 4 Key Laboratory of Breast Cancer Prevention and Therapy, Tianjin Medical University, Ministry of Education, Tianjin, 300060 China; 5 Department of Clinical Laboratory, Tianjin Medical University Cancer Institute and Hospital, National Clinical Research Center for Cancer, Tianjin, 300060 China

**Keywords:** m6A RNA methylation regulator, TCGA, Gene signature, Prognosis, Thyroid carcinoma, Experimental validation

## Abstract

**Background:**

*N*6-Methyladenosine (m6A) is the most common RNA modification and regulates RNA splicing, translation, translocation, and stability. Aberrant expression of m6A has been reported in various types of human cancers. m6A RNA modification is dynamically and reversibly mediated by different regulators, including methyltransferase, demethylases, and m6A binding proteins. However, the role of m6A RNA methylation regulators in thyroid cancer remains unknown. The aim of this study is to investigate the effect of the 13 main m6A RNA modification regulators in thyroid carcinoma.

**Methods:**

We obtained clinical data and RNA sequencing data of 13 m6A RNA methylation regulators from The Cancer Genome Atlas (TCGA) THCA database. We performed consensus clustering to identify the clinical relevance of m6A RNA methylation regulators in thyroid carcinoma. Then we used LASSO Cox regression analysis to generate a prognostic signature based on m6A RNA modification regulator expression. Kyoto Encyclopedia of Genes and Genomes, Gene Ontology and Gene Set Enrichment Analyses were performed to explore differential cellular processes and signaling pathways between the two groups based on risk signature.

**Results:**

We found that most of the m6A RNA modification regulators are down-regulated in 450 patients with thyroid carcinoma. We derived a three m6A RNA modification regulator genes-based risk signature (FTO, RBM15 and KIAA1429), that is an independent prognostic biomarker in patients with thyroid carcinoma. Moreover, we found that this risk signature could better predict outcome in male than female. Functional research in vitro demonstrated that the m6A RNA methylation regulators involved in the model acted significant role in the proliferation and migration of thyroid cancer cells.

**Conclusions:**

Our study revealed the influence of m6A RNA methylation regulators on thyroid carcinoma through biological experiments and three-gene prognostic model.

**Supplementary Information:**

The online version contains supplementary material available at 10.1186/s12935-021-02090-9.

## Introduction

Thyroid carcinoma is one of the most prevalent human malignancies, and its incidence has significantly increased over the past 10 years [1]. According to the histological characteristics, thyroid carcinoma can be classified into four types, including anaplastic, follicular, medullary, and papillary thyroid carcinoma [2]. Although thyroid cancer has been considered a disease with favorable outcome, the patients with advanced thyroid carcinoma have a poor 5-year survival rate [3, 4]. Therefore, it is important to identify effective diagnostic markers, prognostic indicator and therapeutic targets for thyroid carcinoma treatment.

*N*6-Methyladenosine (m6A), a modification occurs at the N6 position of RNA adenine (A), is the most abundant modification on RNA molecules. m6A was first discovered in 1974 [5], occurring in various types of RNAs, including mRNA, tRNA, snRNA, and long noncoding RNA [6]. The m6A methylation is the most prevalent and well recognized modification among all RNA modifications. Similar to DNA methylation, m6A methylation regulates the post-transcriptional expression of target RNAs by affecting RNA alternative splicing, stability, nuclear export, mRNA decay, and translation [7–10]. Its regulatory effects are mediated by the dynamic and reversible interactions among methyltransferases (“writers”), demethylase (“erasers”) and RNA-binding proteins (“readers”), which can add, remove, and recognize m6A-modified sites and generate different functions [11]. Methyltransferases, known as the “writers”, are composed of METTL3/14/, RBM15, WTAP, KIAA1429, and ZC3H13, regulating the process of methylated modification of RNA. Demethylase, which are considered m6A “erasers”, consist of FTO and ALKBH5, mediating the demethylation process of RNA. RNA-binding proteins, known as m6A “readers”, recognize RNA methylated information and participate in the translation and degradation of downstream RNA, including YTHDC1, YTHDC2, YTHDF1, YTHDF2, and HNRNPC. Increasing studies have shown that abnormal m6A modification plays an important role during tumorigenesis and progression in a variety types of human cancers, including oral squamous cell carcinoma [12], acute myeloid leukemia (AML) [13, 14], glioblastoma [15], hepatocellular carcinoma [16], pancreatic cancer [17] and breast cancer [18]. However, the association between m6A RNA modification regulators and thyroid carcinoma remains unknown.

In present study, we identified that 13 were abnormally expressed in thyroid carcinoma tissues compared to those m6A RNA methylation regulators in normal tissues from The Cancer Genome Atlas (TCGA) thyroid carcinoma database. Based on the signature of 13 m6A genes, we divided patients into two clusters by consensus clustering method and compared the clinical outcomes of two clusters. Via Cox univariate analysis, the least absolute shrinkage and selection operator (LASSO) Cox regression analysis was employed to construct a prognostic gene signature with three m6A methylation regulatory genes. The differences in the biological pathways were also explored by Kyoto Encyclopedia Genes and Genomes (KEGG), Gene Ontology (GO) and gene set enrichment analysis (GSEA) between subgroups with different prognosis. Our results emphasized the significance of m6A methylation-related genes in thyroid cancer and established a prognostic gene signature for predicting the prognosis in patients with thyroid cancer. Finally, biological experiments in vitro confirmed that downregulation of the m6A RNA methylation regulators involved in the model could significantly influence the proliferation and migration of thyroid cancer cells.

## Materials and methods

### Dataset

All data were obtained from the TCGA database (https://portal.gdc.cancer.gov/), containing RNA‐seq transcriptome data and the corresponding clinicopathological information on 450 thyroid carcinoma patients (450 cases of tumor tissues and 58 cases of normal tissues; Additional file 1: Table S1).

### Differential expression analysis of m6A RNA methylation regulators

Limma package and Wilcox-test were used for screening the differentially expressed genes (DEGs) between tumor tissues and normal tissues. We extracted the expression matrix of the 13 known m6A RNA methylation regulators, including METTL3, YTHDF1, KIAA1429, YTHDC2, ALKBH5, YTHDF2, YTHDC1, ZC3H13, METTL14, FTO, WTAP, RBM15 and HNRNPC. Heatmap and vioplot were used to display the different expression of the 13 m6A RNA methylation regulators in normal tissues and tumor tissues. Pearson correlation analysis was used to evaluate the association among different m6A RNA methylation regulators with “corrplot” R package.

### Consensus clustering analysis

The TCGA thyroid carcinoma cohort was divided into different clusters by consensus expression of m6A RNA methylation regulators with “ConsensusClusterPlus” R package [19]. Principal component analysis (PCA) was performed by limma and ggplot2 package. Kaplan–Meier survival curves and the log-rank test were used to evaluate patients of thyroid carcinoma with different clusters. Chi-square test was used to compare the distribution of age, gender and stage between different clusters.

### Prognostic signatures construction and prediction

Univariate Cox analysis was performed to assess the prognostic value of m6A RNA methylation regulators. Regulators with a hazard ratio (HR) < 1 or > 1 were considered as protective or risk genes, respectively. The LASSO Cox regression algorithm was used to construct the optimal prognostic model out of the 13 m6A regulators. The risk score for each patient was calculated as follows:

$$Risk\;Score = \sum \limits_{i = 1}^{n} \beta i*Expgene\left( i \right).$$

where *n* is the number of genes in this prognostic model, *β* represents the regression coefficient, and *Expgene(i)* is the expression level of each gene.

We divided the patients from the TCGA thyroid carcinoma cohort into high-risk and low-risk groups based on their risk scores being below or above the median value. The survival difference in overall survival between high-risk group and low-risk group was analyzed by the Kaplan–Meier method with a two-sided log-rank test. Receiver operating characteristic (ROC) curve was constructed to evaluate the accuracy of the model prediction. Chi-square test was performed to compare the differences of clinicopathological features between high-risk group and low-risk group. Heatmaps were generated using the pheatmap R package and used to visualize the differences. Univariate and multivariate Cox regression analyses were used to identify the independent prognostic factors for the TCGA thyroid carcinoma cohort. The survival difference between high-risk group and low-risk group subgrouped by age, gender, tumor size and stage was further evaluated.

### Signaling pathways and cellular processes affected by m6A regulators

GSEA was used to explore the biological pathways underlying the high-risk and low-risk groups defined by the prognostic signature in the thyroid carcinoma patients. KEGG gene sets (v7. 0) and phenotype label (HighRisk vs. LowRisk) files were generated and loaded into the GSEA software (v4.0.3; Broad Institute, Cambridge, MA). The permutation test run 1000 times. The GO and KEGG pathway enrichment analyses were applied for exploring the relevant signaling pathways enriched by DEGs between the high-risk group and the low-risk group using clusterProfiler R package.

### Cell culture

The TPC1 cell line was obtained from American Tissue Culture Collection (Beijing, China) and cultured in RPMI 1640 with 10% fetal bovine serum (FBS, Life Technologies) and 1% penicillin/streptomycin at 37 °C in a 5% CO_2_ incubator.

### Transient transfection and small interfering RNAs (siRNAs)

The siRNA of FTO, RBM15, and KIAA1429 were purchased by RiboBio (Guangzhou, China). Sequences of small interfering RNAs (siRNAs) were listed in Additional file 1: Table S2. When degree of cell fusion reached 40%, the 50 nmol/L siRNA mix were transiently transfected into cells by using FuGENE® HD Transfection Reagent (Promega) according to the manufacturer’s protocol.

### RNA extraction and reverse transcription quantitative PCR

The total RNAs were extracted by TRIzol® Reagent (Life Technologies) after transfection for 48 h, and were reversed to cDNA by EasyScript® All-in-One Frist-Strand cDNA Synthesis SuperMix for qPCR (One-Step gDNA Removal) (Transgen). TansStart® Top Green qPCR Supermix (+DyeII) (Transgen) was performed to test the mRNA expression of FTO, RBM15 and KIAA1429 according to the manufacturer’s instructions. The primer sequences used for RT-qPCR were listed in Additional file 1: Table S3.

### MTT and colony formation assay

For MTT assay, 2 × 10^3^ cells were seeded in 96-well plates after transfection for 48 h. Cell proliferation was assessed by 3-(4,5-dimethylthiazol-2-yi)-2,5-diphenyltetrazolium bromide (MTT) at different time points (1, 2, 3, 4, 5 days) using BioTek microplate reader. In the colony formation assay, 5 × 10^2^ cells were seeded in 6-well plates for 2–3 weeks according to the size of colonies. The colonies were fixed with 4% paraformaldehyde and stained with crystal violet.

### Edu assay

1 × 10^5^ cells were seeded in 24-well plates overnight, and cells were cultured with 200 μL medium with Edu (1:1000) at 37 °C incubator for 2 h. Then cells were washed, fixed, and stained using Cell-LightTM Edu Apollo488 In Vitro Kit (RiboBio, China) according to the manufacture’s instruction. Positive fluorescence Microscope (Zeiss) was used to take pictures.

### Cell cycle

1 × 10^6^ cells were collected and fixed with cooled 75% ethanol for 24 h. After washed with ice-cold PBS two times, the fixed cells were incubated with PI and DNase-free RNase A at room temperature for 20 min. Then the stained cells were applied to cell cycle analysis by flow cytometry (BD FACSCantoII).

### Migration and invasion assay

6 × 10^4^ cells were seeded in the upper chamber with DMEM medium with 10% FBS and the lower chamber contained a medium with 20% FBS. After cultured at 37 °C incubator for 6–10 h, cells in the upper chamber were fixed and stained by three-step set (Thermo Scientific, USA). For the invasion assay, the upper chamber was pre-beaded with 20 μg/well Matrigel (BD Biosciences, USA) at 37 °C incubator, and the other procedure was similar to that of the migration assay. Images of the invaded or migrated cells were captured under a light microscope at 100× magnification. The number of invaded or migrated cells was counted under a microscope in five predetermined fields.

### Wound healing assay

Cells were cultured in serum-free medium in 6-well plates all night when cell fusion approximated to 100% and then wound were drawn by a 10 μL pipette tip at the bottom of 6-well plates. Phosphate Buffer Saline (PBS) was used to wash floating cells and culture medium was refreshed. Wound healing procedure were observed and imaged by a fluorescence microscope at the culturing time of 0, 24 h, respectively.

### Western blot and antibodies

Cellular protein was extracted by RIPA buffer with PMSF (Solarbio, China). The protein concentration was determined by BCA method (Thermo scientific, USA). Protein samples were separated on 10% SDS-PAGE gels, and transferred to PVDF membranes. Then the membranes were blocked in 5% skimmed milk for 2 h at room temperature, and incubated with the diluted primary antibody overnight at 4 °C. The membranes were washed with TBST three times and incubated with secondary antibody for 1 h at room temperature. Finally, the protein binding was detected by ECL (Millipore, USA). The antibodies used for western blot were listed in Additional file 1: Table S4.

### Statistical analysis

Data were presented as means ± SD. Student’s t-test was used to determine the significance of differences between the experimental and control groups. A P value of less than 0.05 was considered statistically significant. All calculations were performed with SPSS software (IBM Corp., USA). Data analysis was performed with the R v3.6.1.

## Results

### Patient demographics and tumor characteristics

The thyroid carcinoma cohort was downloaded from TCGA and the clinicopathological characteristics of patients were listed in Additional file 1: Table S1. Only cases with clinicopathological and prognostic information were included in this study (n = 450).

### The landscape of m6A RNA methylation regulators in thyroid carcinoma

**Fig. 1:**
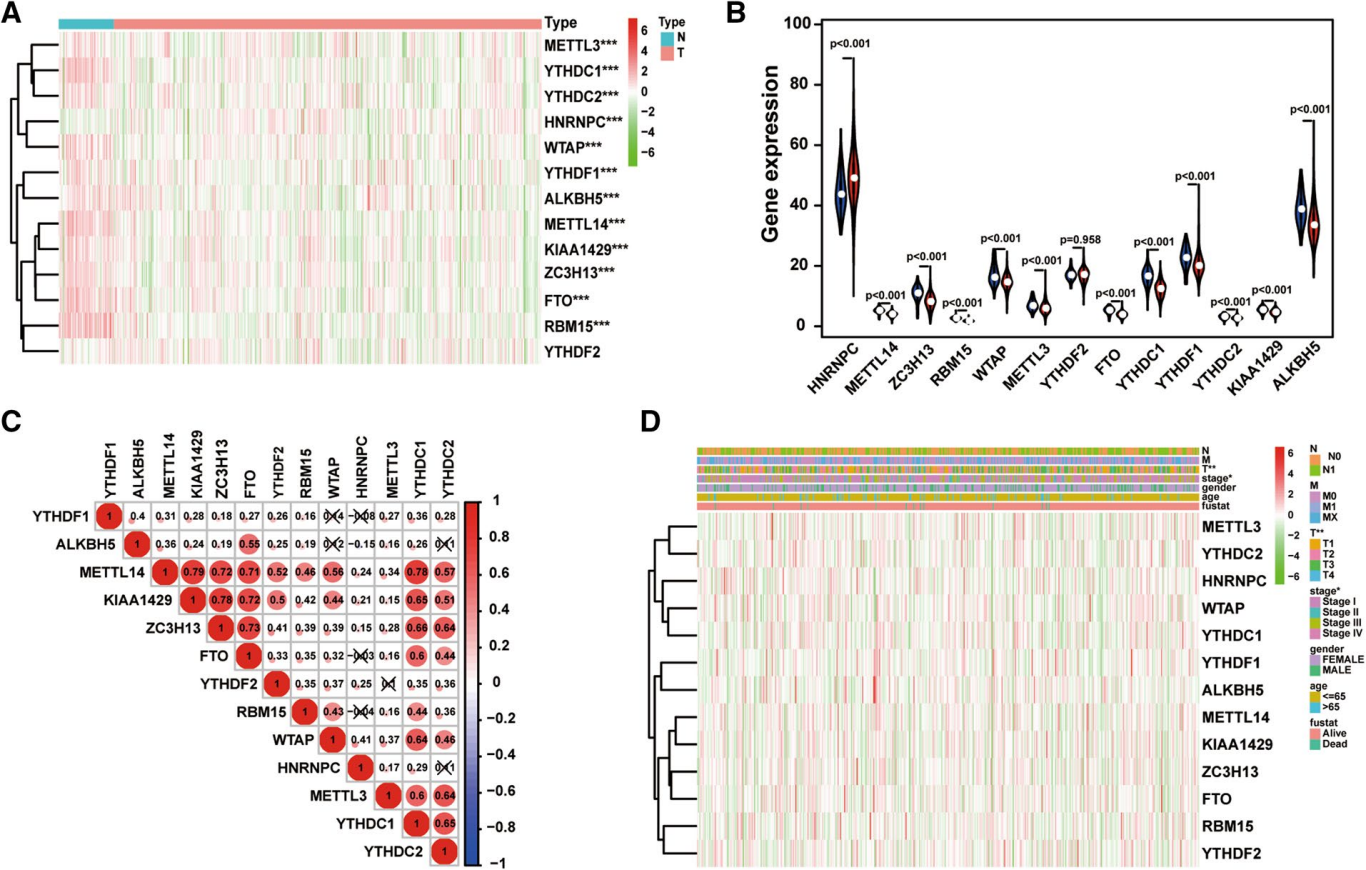
The landscape of m6A RNA methylation regulators in thyroid cancer. **A** The heatmap of the expression levels of m6A RNA methylation regulators in thyroid carcinoma from TCGA database. N, normal. T, tumor. Red, up-regulated. Green, down-regulated. **B** Violet visualizing the differentially m6A RNA methylation regulators in thyroid carcinoma. Blue, normal. Red, tumor. **C** Spearman correlation analysis of the 13 m6A RNA methylation regulators in thyroid carcinoma. **D** The expression levels of m6A RNA methylation regulators in thyroid carcinoma with different clinicopathological characteristics

To investigate the role of m6A RNA methylation regulators in thyroid carcinoma development and progression, we first determined the expression levels of 13 m6A RNA methylation regulators 450 thyroid carcinoma tissues and 58 normal thyroid tissues from TCGA dataset by heatmap (Fig. 1A). We observed that the expression level of 11 m6A RNA methylation regulators (METTL3, METTL14, RBM15, WTAP, YTHDC1, YTHDC2, YTHDF1, KIAA1429, FTO, ZC3H13 and ALKBH5) was significantly decreased in thyroid carcinoma tissues compared to those in normal thyroid tissues, whereas the expression level of HNRNPC was increased in thyroid carcinoma tissues (Fig. 1B). The alternation of m6A RNA methylation regulators ratio is an intrinsic feature that can characterize individual differences [20]. The correlations between 13 m6A RNA methylation regulators were analyzed by Spearman correlation analysis. As shown in Fig. 1C, the relationship between the 13 m6A RNA methylation regulators were strongly correlated. Moreover, the METTL14 gene was most strongly correlated with KIAA1429 and YTHDC1 genes (r = 0.78), and the ZC3H13 gene and the KIAA1429 gene were also most relevant (r = 0.78). In addition, we investigated the association between each individual m6A RNA methylation regulator and the clinicopathological factors of patients with thyroid cancer, including age, gender, stage, tumor size, lymph node status and metastasis and found a robust relationship between m6A RNA methylation regulator and clinicopathological factors of patients with thyroid cancer (Fig. 1D).

### Consensus clustering of m6A RNA methylation regulators identified two subgroups of thyroid carcinoma

**Fig. 2:**
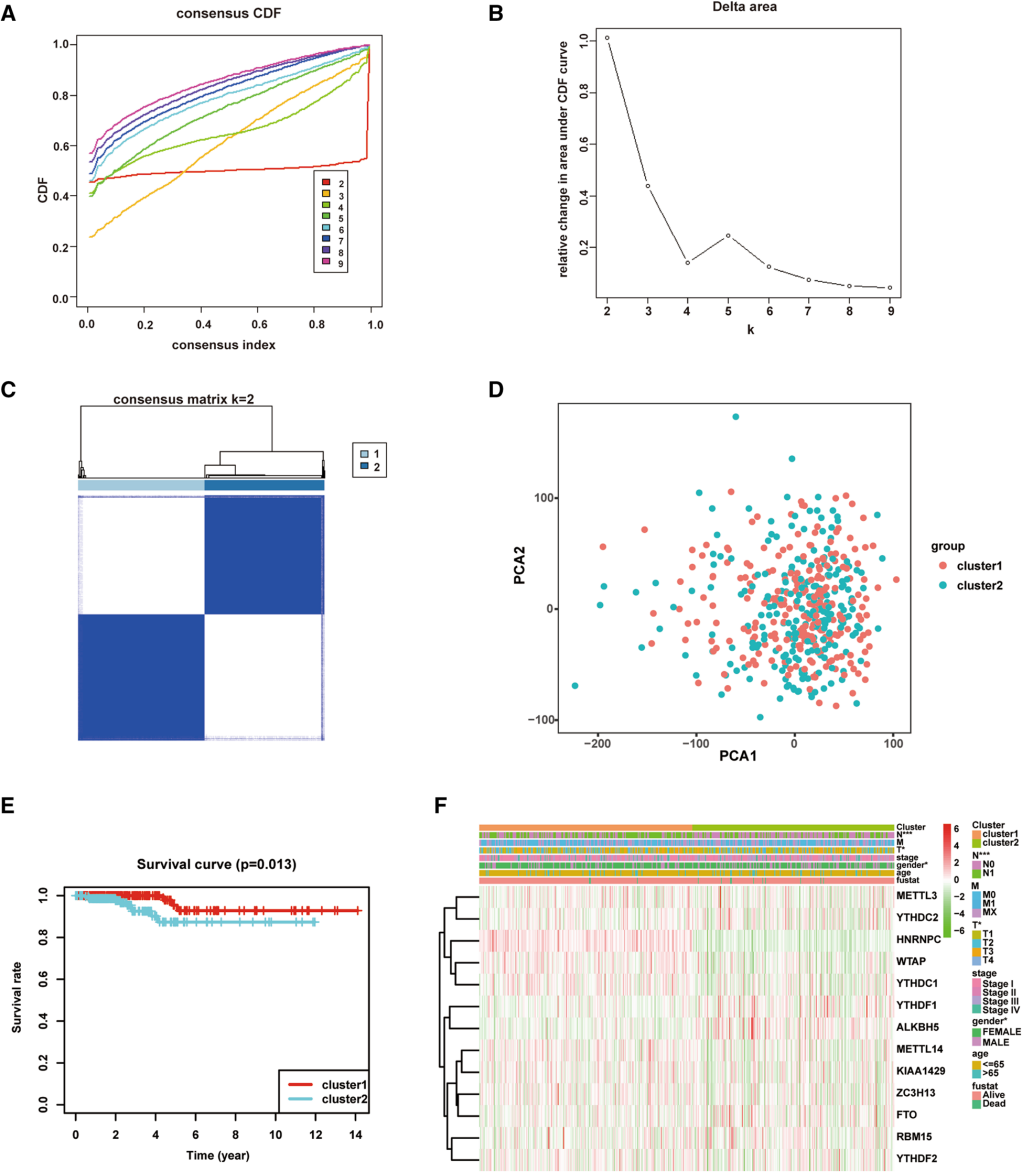
Identification of consensus clusters by m6A RNA methylation regulators. **A** Consensus clustering cumulative distribution function (CDF) for k = 2–9. **B** Relative alteration in area under CDF curve for k = 2–9. **C** Consensus clustering matrix for k = 2. **D** Principal component analysis of the total RNA expression profile from TCGA database. **E** Kaplan–Meier analysis of overall survival for 450 patients with thyroid carcinoma from TCGA database. **F** Heatmap and clinicopathologic characteristics of the two clusters defined by the m6A RNA methylation regulators consensus expression

To provide insight into the clinical relevance of m6A RNA methylation regulators in thyroid carcinoma, we divided the patients into subgroups based on the expression of m6A RNA methylation regulators. “k” was used to represent the number of subgroups (Fig. 2A, B). After consensus clustering analysis, k = 2 was regarded as the most appropriated selection to separate the patients with thyroid carcinoma into two subgroups, namely cluster 1 (n = 231) and cluster 2 (n = 219) (Fig. 2C). To verify our classification, we analyzed the two subgroups by PCA. The results indicated that both cluster 1 subgroup and cluster 2 subgroup could be gathered together (Fig. 2D). Furthermore, we observed that the cluster 1 subgroup had a significantly favorable outcome compared with that in the cluster 2 subgroup (*P* = 0.013; Fig. 2E). There were significant differences in clinicopathological characteristics between the two groups, including gender, tumor status and lymph node status (Fig. 2F).

### Prognostic value of risk signature

**Fig. 3:**
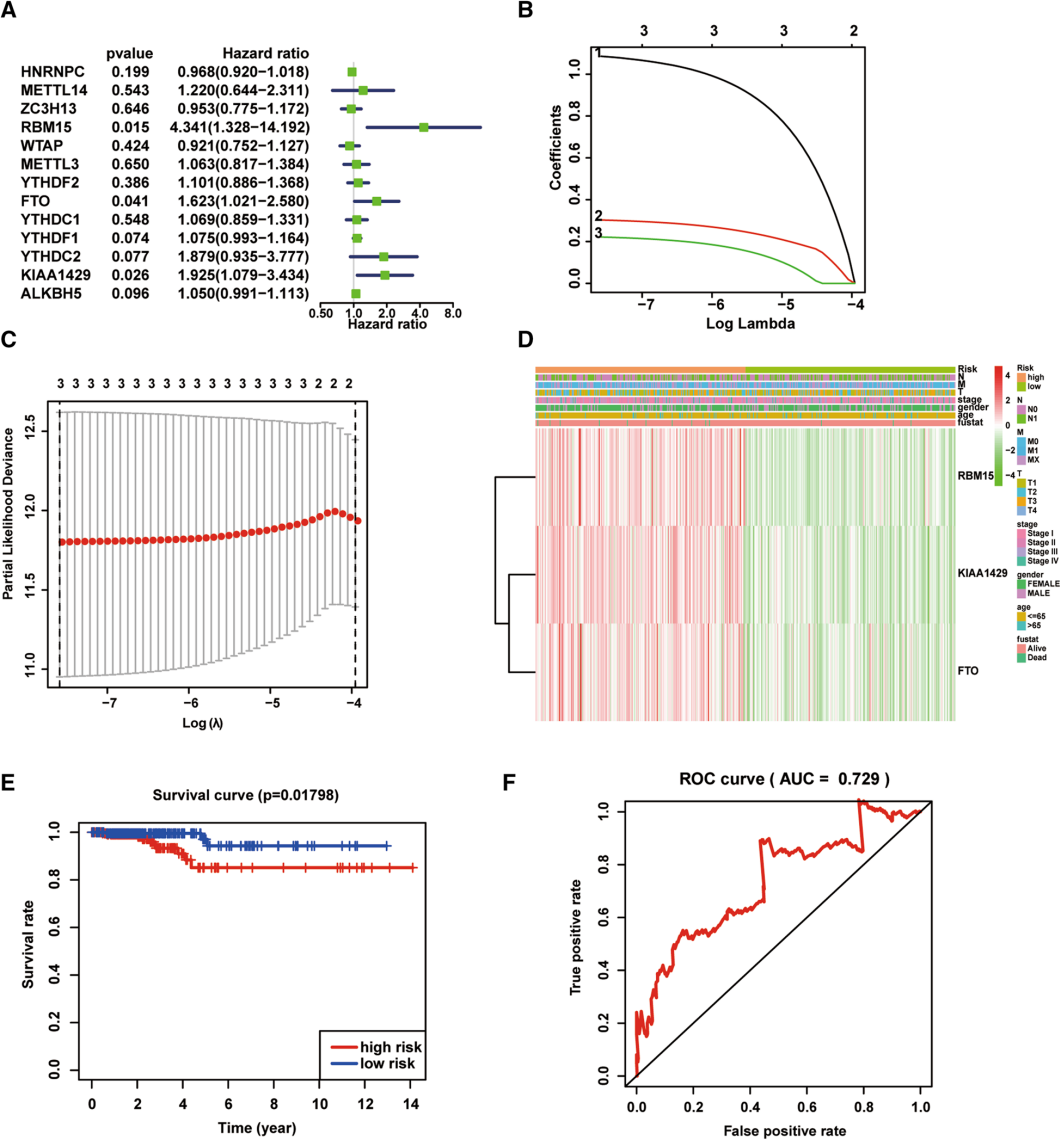
Construction of risk signature with three m6A RNA methylation regulators. **A** Univariate Cox analysis of the 13 m6A RNA methylation regulators to identify the genes that significantly correlated with overall survival. **B**, **C** Coefficients of three m6A RNA methylation regulators calculated by LASSO Cox regression algorithm. **D** The relationship between the three m6A RNA methylation regulators expression and clinicopathologic characteristics in high-/low-risk subgroup of thyroid carcinoma patients. **E** Kaplan–Meier analysis of overall survival in high-/low-risk subgroup of thyroid carcinoma patients. **F** The prediction efficiency of risk signature analyzed by ROC curve

To better understand the prognostic role of the m6A RNA methylation regulators in thyroid carcinoma, we performed a univariate Cox regression analysis on the expression level of the 13 m6A RNA methylation regulators genes. As shown in Fig. 3A, high expression of RBM15 (HR = 4.341, 95% CI = 1.328–14.192, *P* = 0.015), FTO (HR = 1.623, 95% CI = 1.021–2.580, *P* = 0.041) and KIAA1429 (HR = 1.925, 95% CI = 1.079–3.434, *P* = 0.026) had a poor overall survival in patients with thyroid carcinoma. We next applied the LASSO Cox regression algorithm to the m6A RNA methylation regulator genes in the TCGA dataset and constructed an optimal prognostic gene signature. Three genes (RBM15, FTO and KIAA1429) were selected to construct the optimal prognostic gene signature based on the minimum criteria, and the coefficients obtained from the LASSO algorithm were used to calculate the risk score for TCGA dataset (Fig. 3B, C). Based on the median risk score, we separated the thyroid carcinoma patients into high- and low-risk subgroups. Although there was no significant difference in clinicopathological characteristics between the two subgroups (Fig. 3D), the high-risk subgroup had a worse overall survival in patients with thyroid carcinoma (Fig. 3E). The area under the ROC curve (AUC) was also calculated to evaluate the ability of the prediction model (Fig. 3F).

### Investigation of biological pathways affected by m6A RNA methylation regulators

**Fig. 4:**
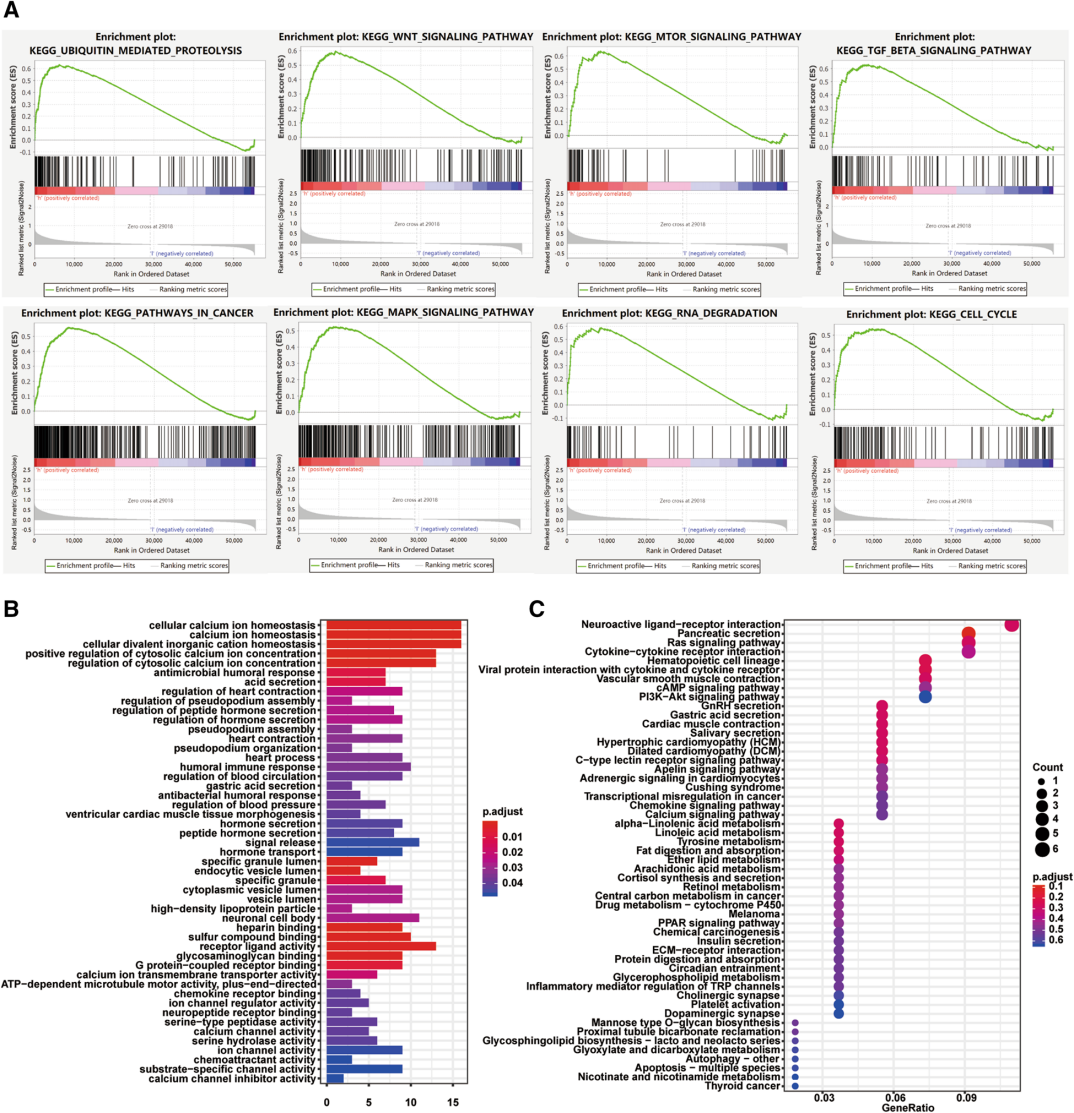
Differential signaling pathways and cellular processes between the high-risk subgroup and the low-risk subgroup defined by a 3-gene expression signature. **A** The highly enriched gene sets in high-risk group analyzed by GSEA. **B** Biological processes of differentially expressed genes between the high-risk subgroup and the low-risk subgroup. **C** KEGG pathways in which differentially expressed genes were distributed

We next explored the biological signaling pathways underpinning the different outcome between the high-risk and low-risk subgroups by GSEA analysis. We observed an increased expression of genes involved in the following biological processes/signaling pathways in the high-risk subgroup, including ubiquitin mediated proteolysis (NES = 2.124, FDR < 0.001), Wnt signaling pathway (NES = 2.115, FDR < 0.001), mTOR signaling pathway (NES = 2.103, FDR < 0.001), TGF-β signaling pathway (NES = 2.059, FDR = 0.002), pathways in cancer (NES = 2.050, FDR = 0.002), MAPK signaling pathway (NES = 2.009, FDR = 0.04), RNA degradation (NES = 1.858, FDR = 0.013), and cell cycle (NES = 1.781, FDR = 0.021, Fig. 4A). A total of 214 differentially expressed genes (log FC > 1 and *P* < 0.05) were identified between the two subgroups and the differentially expressed genes were then annotated using GO (Fig. 4B) and KEGG (Fig. 4C) analyses. Several malignancy-related biological processes or signaling pathways were noticeable, including Ras signaling pathway, phosphoinositide 3‐kinase (PI3K)-AKT signaling, peroxisome proliferator‐activated receptor (PPAR) signaling. The correlation between this three m6A RNA methylation regulators and 214 differentially expressed genes was further identified, and the regulatory network was visualized using Cytoscape software (Additional file 1: Figure S1). CRYBG3 is the most strongly correlated with FTO, and PWAR5 is the most strongly associated with KIAA1429. Nevertheless, the various regulatory networks we explored have not been reported in previous studies.

### The prognostic risk score was an independent prognostic factor in patients with thyroid carcinoma

**Fig. 5:**
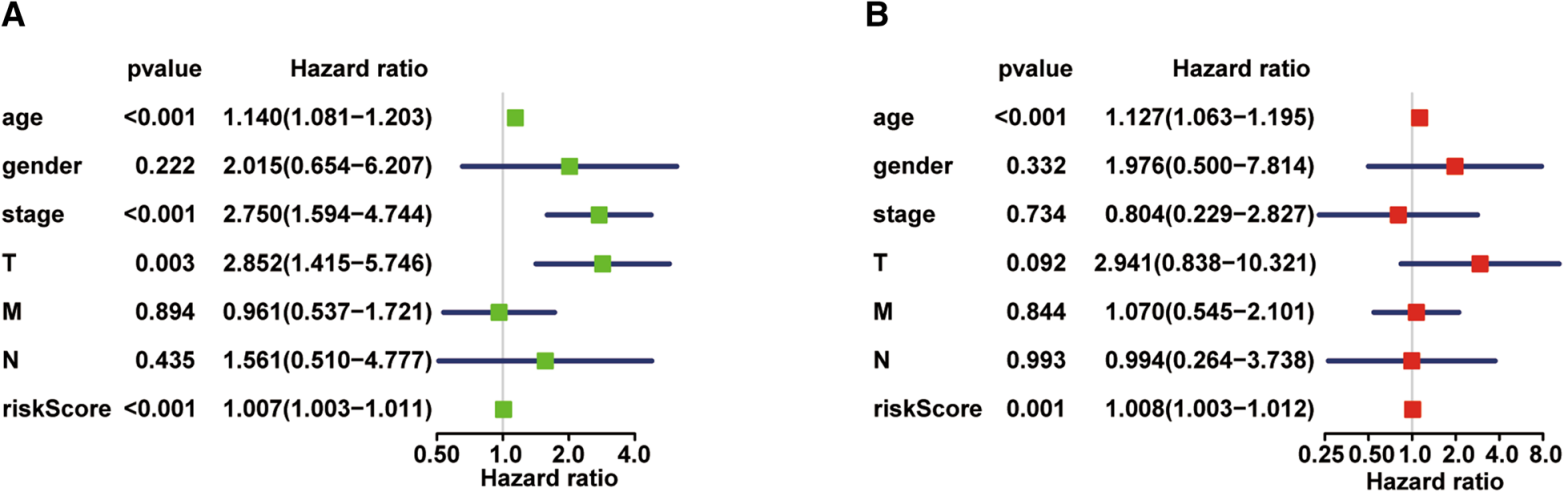
Evaluation of prognostic values of risk scores in patients with thyroid carcinoma. **A** Univariate Cox analyses of the association between clinicopathological characteristics and overall survival in patients with thyroid carcinoma. **B** Multivariate Cox analyses of the association between clinicopathological characteristics and overall survival in patients with thyroid carcinoma

**Fig. 6:**
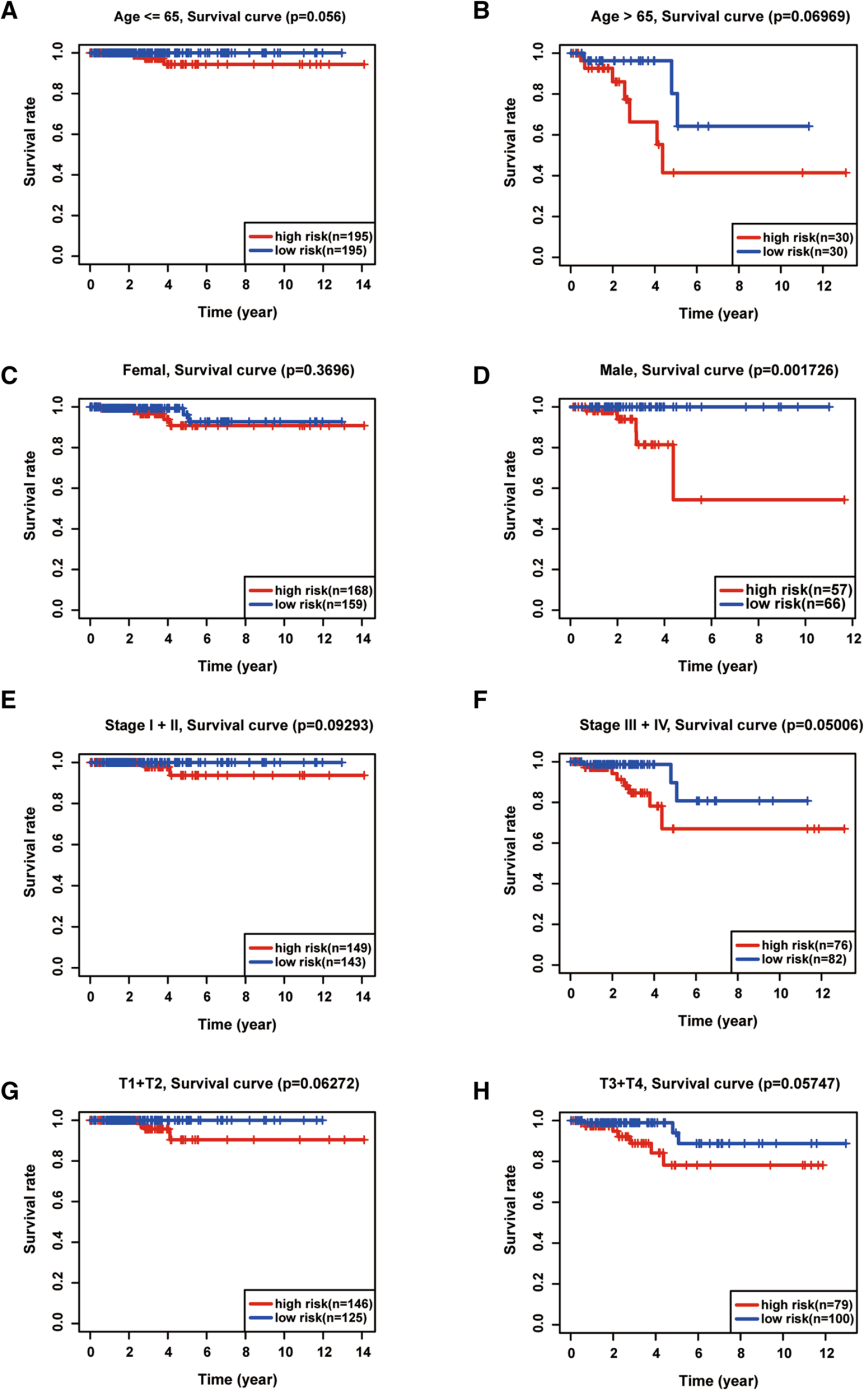
Kaplan–Meier analysis of overall survival in patients with thyroid carcinoma stratified by age (**A**, **B**), gender (**C**, **D**), stage (**E**, **F**) and tumor size (**G**, **H**)

Next, we performed univariate and multivariate Cox regression analyses to determine whether the risk score is an independent prognostic biomarker in patients with thyroid carcinoma. The univariate analysis showed that the age (*P* < 0.001, HR = 1.140, 95% CI = 1.081–1.203), stage (*P* < 0.001, HR = 2.750, 95% CI = 1.594–4.744), tumor size (*P* = 0.003, HR = 2.852, 95% CI = 1.415–5.746), and risk score (*P* < 0.001, HR = 1.007, 95% CI = 1.003–1.011) were significantly correlated with the overall survival (Fig. 5A). Multivariate Cox regression analysis was performed using variables significant in univariate analysis. As shown in Fig. 5B, the age (*P* < 0.001, HR = 1.127, 95% CI = 1.063–1.195) and risk score (*P* = 0.001, HR = 1.008, 95% CI = 1.003–1.012) were identified as the independent prognostic factors in patients with thyroid carcinoma. We also determined whether the risk score are able to predict clinical prognosis in thyroid carcinoma stratified by age (Fig. 6A, B), gender (Fig. 6C, D), stage (Fig. 6E, F) and tumor size (Fig. 6G, H). We found that the risk score could predict outcome better in Male than Female (Fig. 6C, D).

### Downregulation of FTO inhibited proliferation and promoted metastasis in thyroid cancer cell

**Fig. 7:**
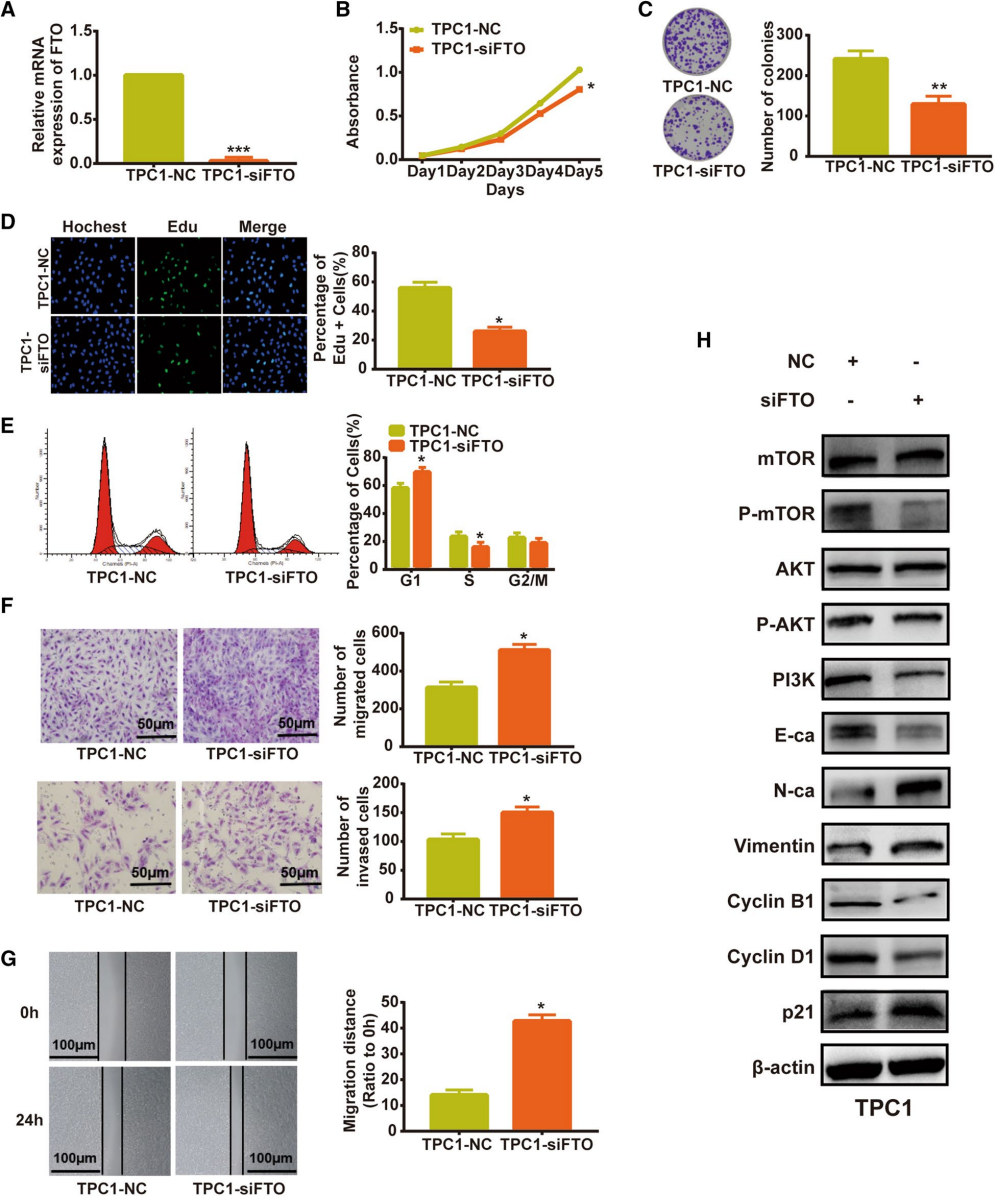
Downregulation of FTO inhibited proliferation and promoted metastasis in thyroid cancer cell. **A** The expression of FTO in indicated cells determined by RT-qPCR. **B**–**E** The effect of FTO on the proliferation of TPC1 cells determined by MTT (**B**), colony formation (**C**), Edu (**D**) and cell cycle (**E**) assays. **F**, **G** The effect of FTO on the migration and invasion of TPC1cells determined by transwell (**F**) and wound healing (**G**) assays. **H** Western blot analysis of the role of FTO in TPC1 cells. The data shown in **A**–**G** are the Mean ± S.D. Student’s t-test for **A**–**G**. *p < 0.05, **p < 0.01, ***p < 0.001

To explore the role of FTO in thyroid cancer in vitro, we transfected the specific siRNA of FTO into thyroid cancer cell TPC1. The interference efficiency was accessed by RT-qPCR (Fig. 7A). MTT and colony formation assays showed that downregulation of FTO inhibited cell growth (Fig. 7B, C). Similarly, FTO silencing inhibited the progression of cell cycle as determined by EDU (Fig. 7D) and cell cycle assays (Fig. 7E). Whereas, silencing of FTO promoted the migration and invasion ability of TPC1 cells (Fig. 7F, G). Additionally, the western results showed that downregulation of FTO decreased the expression of E-cadherin and increased the expression of N-cadherin and vimentin (Fig. 7H). FTO silencing reduced expression of Cyclin D1 and Cyclin B1 and raised expression of p21 (Fig. 7H). Moreover, knockdown of FTO inhibited the PI3K/AKT/mTOR signaling pathway (Fig. 7H).

### Knocking down RBM15 inhibited thyroid cancer cell growth, migration and invasion

**Fig. 8:**
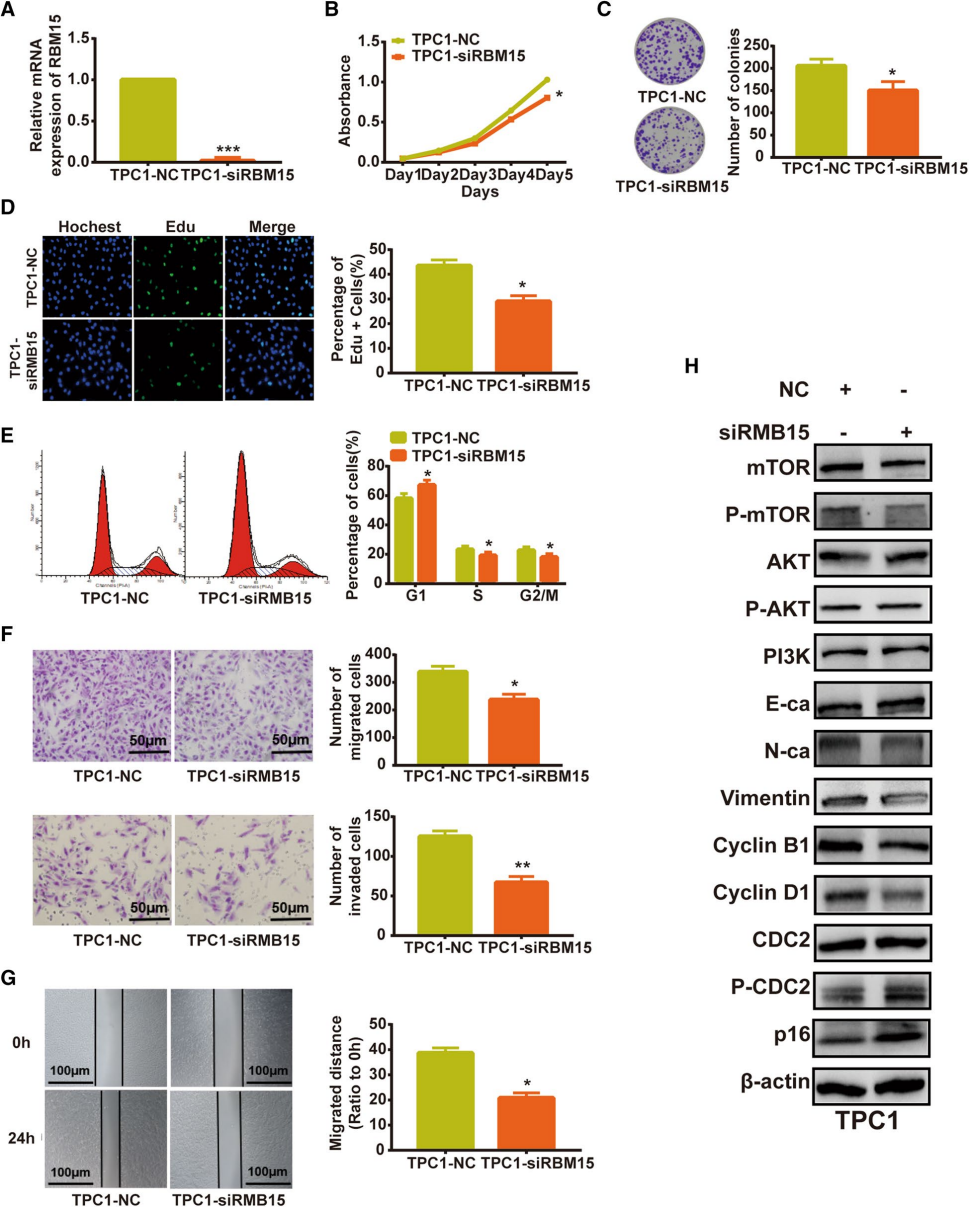
Knocking down RBM15 inhibited thyroid cancer cell growth, migration and invasion. **A** The expression of RBM15 in indicated cells determined by RT-qPCR. **B**–**E** Growth inhibition in TPC1 cells determined by MTT (**B**), colony formation (**C**), Edu (**D**) and cell cycle (**E**) assays. **F**, **G** Cell migration and invasion suppression in TPC1 cells determined by transwell (**F**) and wound healing (**G**) assays. **H** Western blot analysis of the role of RBM15 in TPC1 cells. The data shown in **A**–**G** are the mean ± S.D. Student’s t-test for **A**–**G**. *p < 0.05, **p < 0.01, ***p < 0.001

After knocking down RBM15 in TPC1 cells with specific siRNA (Fig. 8A), cell proliferation was significantly inhibited compared to the control group (Fig. 8B–E), and, the migration and invasion ability of TPC1 cells were also reduced (Fig. 8F, G). Furthermore, western blot analysis showed that silencing RBM15 exhibited decreased expression of Cyclin B1, Cyclin D1, N-cadherin and vimentin as well as increased expression of p16 and P-CDC2 (Fig. 8H). In addition, knockdown of RBM15 inhibited the PI3K/AKT/mTOR signaling pathway (Fig. 8H).

### KIAA1429 promoted thyroid cancer cell proliferation

**Fig. 9:**
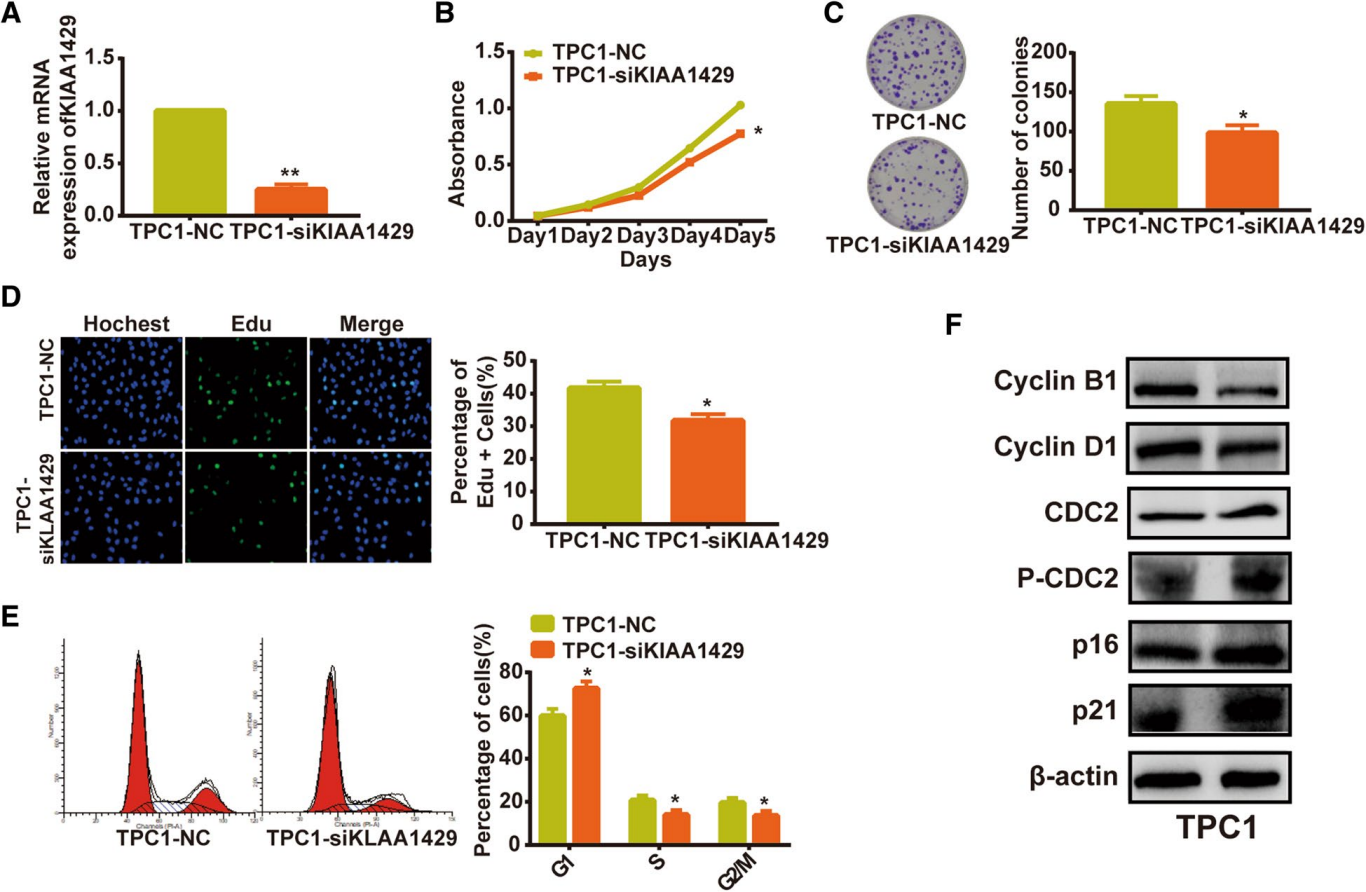
KIAA1429 promoted thyroid cancer cell proliferation. **A** The expression of KIAA1429 in different groups determined by RT-qPCR. **B**–**E** MTT (**B**), colony formation (**C**), Edu (**D**) and cell cycle (**E**) assays showed that knockdown of KIAA1429 inhibited cell proliferation. **F** Western blot analysis revealed that downregulation of KIAA1429 inhibited the progression of cell cycle in TPC1 cells. The data shown in **A**–**E** are the Mean ± S.D. Student’s t-test for **A**–**E**. *p < 0.05, **p < 0.01, ***p < 0.001

We knocked down KIAA1429 expression by using specific siRNA (Fig. 9A), and assessed the tumor cell proliferation abilities with MTT, colony formation assay as well as Edu and cell cycle assays. The results demonstrated that downregulation KIAA1429 significantly suppressed the cell growth and the progression of cell cycle in TPC1 cells (Fig. 9B–E). Meanwhile, the western results indicated that KIAA1429 silencing reduced the expression of Cyclin D1 and Cyclin B1 and increased the expression of p16, p21 and P-CDC2 (Fig. 9F).

## Discussion

m6A, a member of RNA epigenetic modification families, is the most abundant mRNA modification in most eukaryotes and involves almost all steps of RNA metabolism, including splicing, stability, translocation and translation [21]. Although increasing evidence indicates that the m6A RNA methylation regulators act as both oncogene or tumor suppressor in many types of human malignant tumors by regulating the mRNA expression of tumor-related genes, the specific regulatory role of m6A RNA methylation regulators in cancer development and progression remains to be clarified. In the present study, we investigated the role of 13 m6A RNA methylation regulators in thyroid carcinoma and found that the expression of m6A RNA methylation regulators was closely associated with the outcome in patients with thyroid carcinoma.

Analyses of TCGA database revealed that most of m6A RNA methylated regulators except YTHDF2 were dysregulated in thyroid carcinoma, suggesting that m6A RNA modification plays a critical role in thyroid cancer development. Among them, only HNRNPC showed an increased expression in thyroid carcinoma tissues compared with that in normal tissues. HNRNPC, as the “readers” of m6A RNA modification, is well recognized for its regulatory roles in RNA splicing, sequence-unspecific RNA exportation, stability and translation. Abnormal increased expression of HNRNPC was observed in multiple types of tumors, including breast cancer [22], lung cancer [23], glioblastoma [24], and hepatocellular carcinoma [25]. Consistent with their work in other cancers, our results suggest that HNRNPC functions as an oncogene in thyroid carcinoma.

We identified two subgroups of thyroid carcinoma by consensus clustering based on the expression of the m6A RNA methylation regulators, and the cluster 2 subgroup associated with a poorer outcome. In addition, we constructed a risk signature by using 3 m6A RNA methylation regulators (FTO, RBM15, and KIAA1429) and the risk score is an independent prognostic biomarker in patients with thyroid carcinoma. To explain the different prognosis of the two groups, we attempted to explore the mechanisms through GSEA and KEGG analyses. GSEA indicated that the significantly enriched gene sets in the high-risk subgroup with poor survival were some crucial signaling pathways and cellular processes, including ubiquitin mediated proteolysis, Wnt signaling pathway, mTOR signaling pathway, TGF-β signaling pathway, pathways in cancer, MAPK signaling pathway, RNA degradation, and cell cycle. KEGG analysis of differentially expressed genes between the two groups indicated that the m6A RNA methylation regulators were involved in several cancer-related signaling pathways, including PI3K-Akt signaling pathway and PPAR signaling pathway.

FTO is the first mammalian RNA m6A demethylase [26], which belongs to the AlKB family [27]. Evidences have shown that FTO involves in the process of malignant tumors. FTO promotes AML carcinogenesis by enhancing the stability MYC and CEBPA mRNA [28]. In cervical cancer, FTO enhances the chemoradiotherapy-resistance by reducing the m6A modification of β-catenin [29]. Recently, bioinformatic analysis has shown that FTO acts as anti-oncogene in thyroid cancer [30]. KIAA1429, known as VIRMA, mainly regulates the m6A mRNA methylation in 3′ UTR and near stop codon [31]. It is reported that KIAA1429 promotes breast cancer progression by regulating CDK1 [32]. RBM15 was originally described as a 5′ translocation partner of the MAL gene in t (1; 22) (p13; q13) infant acute megakaryocytic leukemia. Thus, RBM15 plays an important role in hematopoietic development, and c-MYC is a potential target of RBM15 in the regulation of adult hematopoietic stem cell and megakaryocyte development [33, 34]. In chronic myelogenous leukemia (CML), RBM15 promotes CML carcinogenesis through its effect on the Notch signalling [35]. However, there are lacking of experiments both in vitro and vivo to validate the role of these genes in the carcinogenesis of thyroid carcinoma. In our study, we first verify the effect of FTO, RBM15 and KIAA1429 on the progression of thyroid carcinoma in vitro. We found that FTO promotes the proliferation and inhibits metastasis in thyroid cancer cell lines. In addition, we proved that RBM15 functions as oncogene in thyroid cancer via PI3K-AKT-mTOR signaling pathway. Moreover, we indicated that KIAA1429 accelerates the progression of thyroid cancer cell proliferation.

## Conclusions

In summary, we determined the expression, potential function and prognostic value of the m6A RNA methylation regulators in thyroid carcinoma. Moreover, we applied the experiments in vitro to identify the effect of prognostic genes on the progression of thyroid cancer. Therefore, our study provides important evidence that the prognostic model was an independent prognostic factor in patients with thyroid carcinoma.

## Supplementary Information

**Additional file 1: Figure S1.** The regulatory network between m6A RNA methylation regulators and differentially expressed genes visualized by Cytoscape softwore. **Table S1.** Characteristics of patients with thyroid carcinoma from TCGA database. **Table S2.** Target sequence of siRNA. **Table S3.** Primers used for RT-qPCR. **Table S4.** Antibodies used for western blot.

## Data Availability

All data generated or analyzed during this study are included in this published article and its Additional file.

## Abbreviations

m6A: *N*^6^-Methyladenosine
THCA: Thyroid carcinoma
TCGA: The Cancer Genome Atlas
KEGG: Kyoto Encyclopedia of Genes and Gene Ontology
GO: Gene ontology
GSEA: Gene Set Enrichment Analyses
LASSO: Least Absolute Shrinkage and Selection Operator
ROC: Receiver operating characteristic
K-M: Kaplan–Meier
OS: Overall survival
DFS: Disease-free survival
HR: Hazard ratio
