# Supplementary material for: The functions and prognostic values of m6A RNA methylation regulators in thyroid carcinoma
Source: Cancer Cell Int. 2021 Jul 19;21:385. doi: 10.1186/s12935-021-02090-9 (PMC8287668; doi:10.1186/s12935-021-02090-9)
Supplement: Supplementary file 1 — Additional file 1: Figure S1. The regulatory network between m6A RNA methylation regulators and differentially expressed genes visualized by Cytoscape softwore. Table S1. Characteristics of patients with thyroid carcinoma from TCGA database. Table S2. Target sequence of siRNA. Table S3. Primers used for RT-qPCR. Table S4. Antibodies used for western blot. [file 12935_2021_2090_MOESM1_ESM.docx]

**
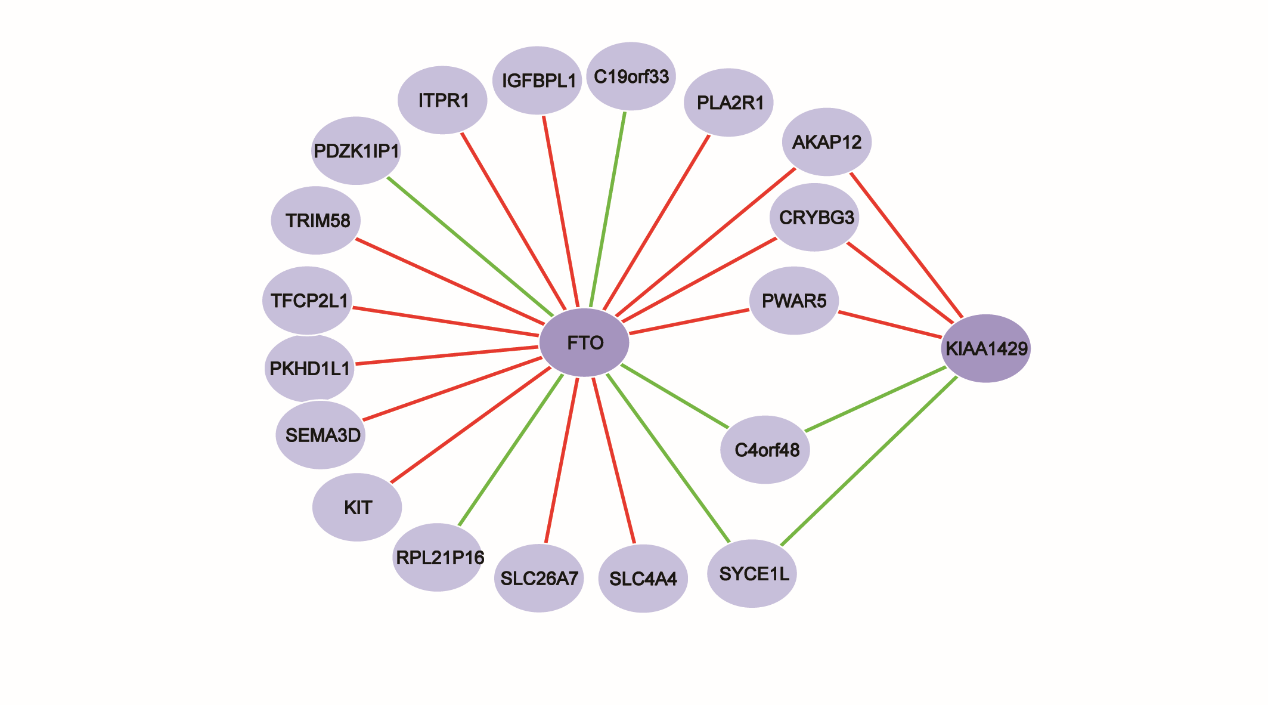
**

**Figure S1.** The regulatory network between m6A RNA methylation regulators and differentially expressed genes visualized by Cytoscape softwore. Red line: positive correlation. Green line: negative correlation.

**Table S1.** Characteristics of patients with thyroid carcinoma from TCGA database

| **Clinicopathological Factors** | **Number of patients**  **(n = 450)** | **%** |
| --- | --- | --- |
| **Age (year)** |  |  |
| < = 65 | 390 | 86.67 |
| > 65 | 60 | 13.33 |
| **Gender** |  |  |
| Female | 327 | 72.67 |
| Male | 123 | 27.33 |
| **Stage** |  |  |
| I | 249 | 55.33 |
| II | 43 | 9.56 |
| III | 105 | 23.33 |
| IV | 53 | 11.78 |
| **Tumor status** |  |  |
| T1 | 131 | 29.11 |
| T2 | 140 | 31.11 |
| T3 | 157 | 34.89 |
| T4 | 22 | 4.89 |
| **Lymph node status** |  |  |
| N0 | 228 | 50.67 |
| N1 | 222 | 49.33 |
| **Metastasis** |  |  |
| M0 | 272 | 60.44 |
| M1 | 7 | 1.56 |
| MX | 171 | 38.00 |

**Table S2** Target sequence of siRNA

| **Target gene (human)** | **Target sequence (5’ to 3’)** |
| --- | --- |
| siFTO 001 | CCTGAACACCAGGCTCTTT |
| siFTO 002 | GGATGACTCTCATCTCGAA |
| siFTO 003 | GTCACGAATTGCCCGAACA |
| siRBM15 001 | GTCCCAGCTTAGTGACGAA |
| siRBM15 002 | GTAGCCGCTTGCATAGTTA |
| siRBM15 003 | GCACGAGAATTTGATCGAT |
| siKIAA1429 001 | GTATCATCTTCTCTTAAGT |
| siKIAA1429 002 | GGATGCTCCTCATAGAGAA |
| siKIAA1429 003 | GGACATGCGTGTTCCTTCA |

**Table S3** Primers used for RT-qPCR

| **Gene name** | **Forward Primer (5’ to 3’)** | **Reverse Primer (5’ to 3’)** |
| --- | --- | --- |
| FTO | TGGTGTCCCAAGAAATCGTG | TGCAGGCCGTGAACCAC |
| RBM15 | TCCCACCTTGTGAGTTCTCC | GTCAGCGCCAAGTTTTCTCT |
| KIAA1429 | CTTGGCAAGTGGCTTGAACC | ACGTAAGGCAGTGGTAAGGC |

**Table S4** Antibodies used for western blot

| **Antibodies** | **Source** | **Company** |
| --- | --- | --- |
| mTOR | Rabbit | Cell signaling technology |
| P-mTOR | Rabbit | Immunoway |
| AKT | Rabbit | Cell signaling technology |
| P-AKT | Rabbit | Cell signaling technology |
| PI3K | Rabbit | Cell signaling technology |
| Vimentin | Rabbit | Immunoway |
| E-cadherin | Rabbit | Proteintech |
| N-cadherin | Rabbit | Proteintech |
| Cyclin B1 | Mouse | Santa cruze |
| Cyclin D1 | Mouse | Santa cruze |
| Beta-actin | Mouse | Cell signaling technology |
| CDC2 | Mouse | Santa cruze |
| P-CDC2 | Mouse | Cell signaling technology |
| P16 | Mouse | Santa cruze |
| P21 | Mouse | Santa cruze |
